# Gene expression pattern in swine neutrophils after lipopolysaccharide exposure: a time course comparison

**DOI:** 10.1186/1753-6561-5-S4-S11

**Published:** 2011-06-03

**Authors:** Gema Sanz-Santos, Ángeles Jiménez-Marín, Rocío Bautista, Noé Fernández, Gonzalo M Claros, Juan J Garrido

**Affiliations:** 1 Grupo de Genómica y Mejora Animal, Departamento de Genética, Facultad de Veterinaria, Universidad de Córdoba, Campus de Rabanales, Edificio Gregor Mendel C5, 14071 Córdoba, Spain; 2 Plataforma Andaluza de Bioinformática, Universidad de Málaga, Edificio de Bioinnovación, C/Severo Ochoa 34, Parque Tecnológico de Andalucía, 29590 Málaga, Spain

## Abstract

**Background:**

Experimental exposure of swine neutrophils to bacterial lipopolysaccharide (LPS) represents a model to study the innate immune response during bacterial infection. Neutrophils can effectively limit the infection by secreting lipid mediators, antimicrobial molecules and a combination of reactive oxygen species (ROS) without new synthesis of proteins. However, it is known that neutrophils can modify the gene expression after LPS exposure. We performed microarray gene expression analysis in order to elucidate the less known transcriptional response of neutrophils during infection.

**Methods:**

Blood samples were collected from four healthy Iberian pigs and neutrophils were isolated and incubated during 6, 9 and 18 hrs in presence or absence of lipopolysaccharide (LPS) from *Salmonella enterica* serovar Typhimurium. RNA was isolated and hybridized to Affymetrix Porcine GeneChip^®^. Microarray data were normalized using Robust Microarray Analysis (RMA) and then, differential expression was obtained by an analysis of variance (ANOVA).

**Results:**

ANOVA data analysis showed that the number of differentially expressed genes (DEG) after LPS treatment vary with time. The highest transcriptional response occurred at 9 hr post LPS stimulation with 1494 DEG whereas at 6 and 18 hr showed 125 and 108 DEG, respectively. Three different gene expression tendencies were observed: genes in cluster 1 showed a tendency toward up-regulation; cluster 2 genes showing a tendency for down-regulation at 9 hr; and cluster 3 genes were up-regulated at 9 hr post LPS stimulation. Ingenuity Pathway Analysis revealed a delay of neutrophil apoptosis at 9 hr. Many genes controlling biological functions were altered with time including those controlling metabolism and cell organization, ubiquitination, adhesion, movement or inflammatory response.

**Conclusions:**

LPS stimulation alters the transcriptional pattern in neutrophils and the present results show that the robust transcriptional potential of neutrophils under infection conditions, indicating that active regulation of gene expression plays a major role in the neutrophil-mediated- innate immune response.

## Background

Neutrophils play a key role in innate immune response. They initiate phagocytosis, degranulation and killing without new synthesis of proteins. However, it has been demonstrated that new gene transcription and protein synthesis are required to maintain full capacity for human neutrophil phagocytosis and associated bactericidal activity [1,2].

LPS treatment enhances neutrophil bactericidal activity, with an alteration in adhesion, respiratory burst, degranulation and motility [3,4]. Thus kinetic study of swine neutrophil response to LPS represents an in vitro model to investigate the innate immune response during bacterial infection.

To test the neutrophil transcriptional potential, global gene expression analysis was performed and the results indicated that the LPS-treated neutrophils increase their transcriptional activity by altering genes involved in different cellular processes including transcriptional regulation, cell signalling, cytoskeletal reorganization, etc. Furthermore, inhibition of apoptosis-related functions may indicate an increase of the neutrophil life span at 9 hr post LPS stimulation. Taken together, our results show that (1) relevant changes occurred in the pattern of gene expression of porcine neutrophils after LPS exposure, and (2) the biological functions identified to be meaningful using Ingenuity Pathway Analysis tool (IPA) suggests that these transcriptional changes play an important role in inflammation and immune response associated with LPS stimulation.

## Methods

### Isolation and stimulation of neutrophils

Blood samples were collected from 4 healthy Iberian pigs at the local abattoir. Neutrophils were isolated with dextran and ficoll sedimentation as previously described [5] and adapted for swine neutrophils. Isolated neutrophils were more than 99% pure and < 1% monocytes were present. Freshly purified neutrophils, ~200 x 10^6^ for each time point, were suspended at 5 x 10^6^ ml^-1^ in 40 ml RPMI medium (Lonza, Basel, Switzerland) supplemented with 10% heat-inactivated bovine fetal serum. Stimulation was performed in 50 ml Falcon tubes and rotated continuously at 37°C for 6, 9 and 18 hr in presence or absence of 100 ng/ml of LPS from *S.* Typhimurium. Neutrophil activation was checked by measuring the released IL-8 in the culture medium by ELISA. The manufacturer certified all reagents (serum, buffers, media and containers) as nonpyrogenic.

### RNA purification and microarray hybridizations

RNA was isolated with RNeasy columns (Qiagen, Valencia, CA) based on the manufacturer´s protocol. Eluted RNA was treated with RNase-Free DNase Set (Qiagen, Valencia, CA) and the integrity, quality and quantity of RNA were checked in the Agilent Bioanalyser 2100 (Agilent Technologies, Palo Alto, CA) and NanoDrop1000 Spectrophotometer (Thermo Scientific, Wilmington, DE).

Biotinylated cRNA was obtained following the Two-Cycle Eukaryotic Target Labeling System from Affymetrix. The labeled cRNA is then cleaned up, fragmented, and hybridized to Affymetrix Porcine GeneChip according to the manufacturer´s procedures (Expression Analysis Technical Manual, Affymetrix Inc., Santa Clara, CA). Chips were scanned with an Affymetrix GeneChip Scanner 3000 (Affymetrix Inc., Santa Clara, CA).

### Data analysis

All data were normalized using the *affy* library of the Bioconductor package (Gentleman et al., 2004) for R (2.10) and adapted in-house scripts. Features appearing in all replicates and having a signal of 50 over background were normalized using robust multi-array analysis (RMA) [6]. Genes were filtered for based on Affymetrix's present/marginal/absent (PMA) calls using mismatch probe intensity, the ratio of PM to MM, only those probes present in at least 80 % of the slide was used in the analysis of microarray. Differential expression and clustering was performed with Spotfire DecisionSite v 9.0 (SP2) as follows: feature values were obtained by a one-way analysis of variance (ANOVA) with a *p*-value < 0.05 and hierarchically clustered using UPGMA method and Pearson´s correlation as the similarity measure. Since the Affymetrix Porcine GeneChip® is not fully annotated in all the features, it was re-annotated with Blast2GO [7] with a minimum E-value of 10-10 and a minimum similarity of 50%.

### Biological interpretation

The networks, functional analysis and canonical pathways were generated through the use of Ingenuity Pathway Analysis (Ingenuity Systems^®^, http://www.ingenuity.com). IPA provides computational algorithms to identify and generate significant biological networks and pathways that are particularly enriched with our genes of interest. Networks are ranked by a score that takes into account the number of focus genes and the size of the networks, indicating the likelihood of the focus genes in a network being found together by chance. The higher the score (score = -log(p-value)), the lower the probability of finding the observed Network Eligible Molecules in a given network by chance. IPA also gives information on biological functions and canonical pathways. Thus, DEG were grouped into known biological functions, networks and canonical pathways based on human and rodent studies.

## Results and discussion

### Changes in gene expression profile in neutrophils stimulated with LPS

**Figure 1:**
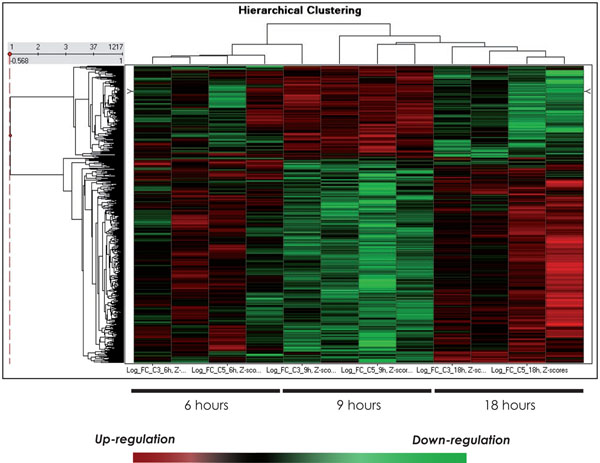
**Hierarchical clustering of differentially expressed genes after LPS exposure**. Heat map were performed with Spotfire Decisionsite v 9.0 (SP2) software. Rows correspond to different genes and the columns represent replicate samples for each time course point. The scale below the heat map indicates the relative changes in gene expression represented by a range of colour.

**Figure 2:**
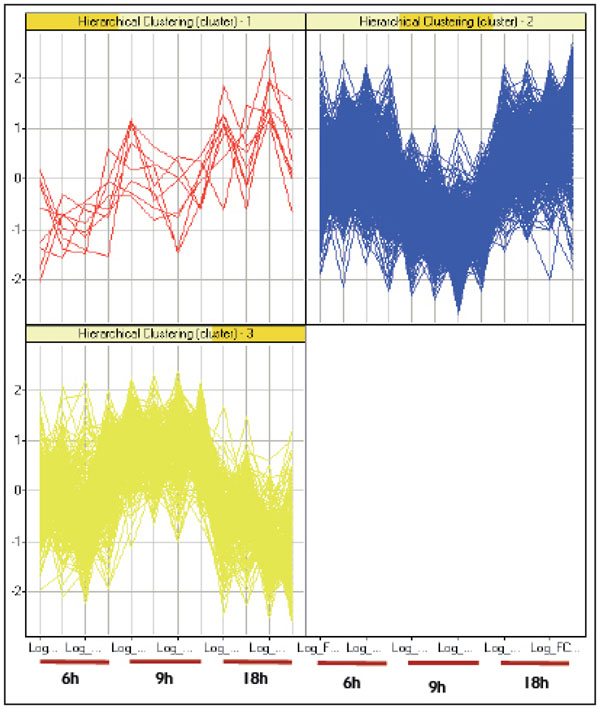
**Differentially expressed genes grouped into three different clusters.** Cluster 1 contains 8 genes with up-regulation tendency through the time course. 747 genes belonging the cluster 2, with a down-regulation tendency at 9 hr. Opposite tendency can be observed in the cluster 3, where 335 genes show an up-regulation at 9 hr and down-regulation at 18 hr.

**Figure 3:**
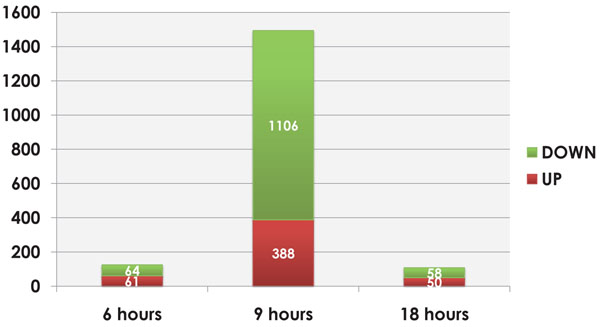
**Differentially expressed genes in each time point**. 125 and 108 genes were altered at 6 and 18 hr respectively, with a similar number of up and down-regulated genes. Most significant transcriptional changes were observed at 9 hr post LPS stimulation. 1106 genes were down-regulated and 388 were up-regulated.

In this study, we performed microarray analysis in order to elucidate the response of the porcine neutrophils to LPS from *S.* Typhimuriun along a time course of 6, 9 and 18 hr *in vitro* stimulation. To achieve that, ANOVA adapted for time course experiments was used, in order to identify genes whose temporal expression patterns differ between control and LPS-treated samples. As a result, 1090 genes were differentially expressed along the time course and the resulting hierarchical clustering is shown in Figure 1. Moreover, genes were clustered according to idealized expression patterns. The query gene is assigned to a cluster designated by the idealized pattern that has the maximal correlation with that gene. Figure 2 shows the three gene expression clusters obtained in this analysis. This criterion for classifying genes according to their similarity to an idealized expression pattern allows us to determine the gene expression tendency through the time course. Clustering results reveal distinct temporal patterns and the most significant transcriptional changes happened after 9 hr post LPS exposure. Individual ANOVA tests for each time point confirm this finding as shown in Figure 3. Thus, at 6 and 18 hr, there are 125 and 108 DEG, respectively, whereas at 9 hr, there are 1494 DEG.

### Biological analysis with Ingenuity Pathway Analysis

IPA identified significant networks, top functions and canonical pathways associated with the DGE for the time course analysis. Networks with score higher than 25 (except for cluster 1) are presented in Additional file 1. Focus genes are shown in bold. Top biofunctions and canonical pathways are listed in Additional file 2.

Cluster 1 contains 8 genes with tendency for up-regulation through the time course, including genes coding for products involved in metabolism (GRHPR, SLC38A1) and immune-related functions, such as adhesion, cellular movement and phagocytosis (SIRPA, SMARCA4) or linked to glucocorticoid signaling (HSPA14), which is known to be implicated in inflammatory response and apoptosis [8].

Cluster 2 includes 747 down-regulated genes at 9 hr. These molecules are involved in cellular organization, molecular transport and protein trafficking and are related to canonical pathways such as JAK/STAT signaling, Cdc42 signaling and protein ubiquitination. Network 3 (Additional file 3) is related to molecular transport, protein trafficking and cellular development. This network is focused on TGFB, a cytokine implicated in process such as neutrophil locomotion, communication among immune cells or glucocorticoid receptor signaling [9].

Finally, cluster 3 consists of 335 up-regulated genes. Functions associated with these molecules are related to cellular assembly and reorganization, cellular maintenance and gene expression. Canonical pathways are related to protein ubiquitination signaling, PDGF signaling and IL-3 signaling which is involved in cell survival by activation of JAK/STAT signaling and BCL2 [10]. Network 2 (Additional file 4) highlights NF-κB interactions and covers several canonical pathways such as acute phase response signaling and interferon signaling.

### Inhibition of spontaneous apoptosis at 9 hrs

**Figure 4:**
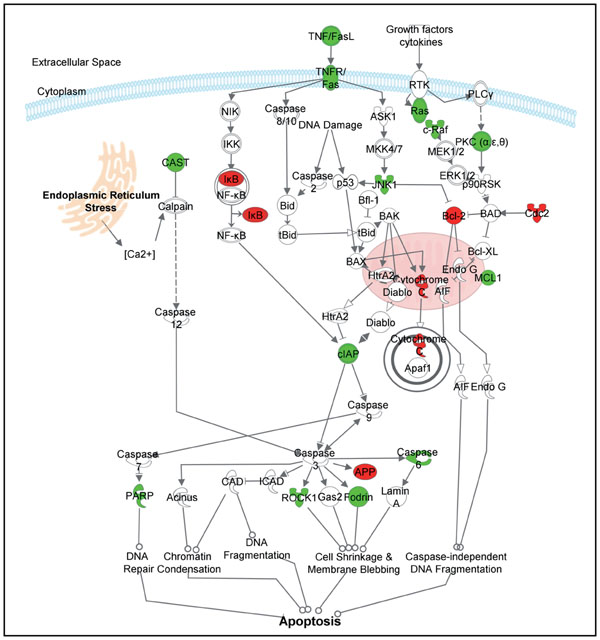
**Delay in apoptosis at 9 hr**. Canonical pathway obtained with IPA for apoptosis signaling. Up and Down –regulated genes are in red and green, respectively.

Turnover of aging neutrophils occurs in the absence of activation through a process known as spontaneous apoptosis [11] and this ability of aged neutrophils to undergo apoptosis plays a central role in resolution of the acute inflammatory response. A well-described effect of LPS is its ability to inhibit spontaneous neutrophil apoptosis [12,13]. Thus, a delay of the apoptosis function could be related to the down-regulation of apoptosis mediators such as CASP6, ROCK1, CFLAR and DIABLO (Figure 4) shown at 9 hr post stimulation. In addition, BCL2, an anti-apoptotic member of the Bcl-2 family of apoptosis regulator proteins [14] was up-regulated at 9 hr.

MCL1 encodes myeloid leukemia cell differentiation protein, Mcl-1, that also belongs to the Bcl-2 family. Alternative splicing occurs at this locus and two transcript variants have been identified. The longer gene product (isoform 1) enhances cell survival whereas the shorter gene product (isoform 2) promotes apoptosis [15]. MCL1 is down-regulated at 9 hr suggesting that it could be the shorter isoform of the Mcl-1 protein.

Also, there is a down-regulation of transcripts coding for some anti-bacterial products like NCF1, NCF2 and NCF4. The reactive oxygen system is autocytotoxic for neutrophils and its down-regulation may contribute to the prolongation of life span during inflammation. Therefore, many components of cell survival pathways are regulated by LPS, and these gene products may be involved in maintaining neutrophil viability. Inhibition of neutrophil apoptosis may improve the host defense against infection by increasing functional longevity of the cells at the inflammatory sites. The results of this study indicate that activated neutrophils not only generate cytokines or phagocytic-related response but also changes in the expression of genes coding for receptors, transcription factors, chromatin remodeling, cytoskeleton reorganization and apoptosis-related genes.

## Competing interests

The authors declare that they have no competing interests.

## Authors' contributions

GS carried out the experimental and IPA work with AJM. RB, NF and GC conducted the statistical analysis of microarray data. JJG conceived, coordinated and supervised the research. GSS drafted the manuscript which was approved by all the authors.

## Supplementary Material

Additional file 1 **IPA Networks** Networks are selected if their scores are higher than 25 for each cluster (except for cluster 1, because the number of genes is too small to analyze with IPA). The table contains columns with the network number, the name of the comparison list, names of the genes involved in the network, the score value and the top functions. Focus genes are shown in bold. Click here for file

Additional file 2 **IPA Biofunctions and Canonical Pathways** Top 10 IPA biofunctions and canonical pathways. a) Biofunctions: Tested by the Fisher Exact test *p*-value. b) Canonical Pathway. For each list, the pathways are ranked by the score [score = -log(*p*-value)] using the same criterions than biofunctions. The table includes the ratio (number of focus molecules in a given pathway divided by the total number of the molecules that conform that pathway) Click here for file

Additional file 3 **Network 3 from gene cluster 2** The top functions of this network are Molecular transport, protein trafficking and cellular development. This network is focused on TGFB1 molecule (orange circle). Canonical pathways and functions are overlaid with red and yellow edges, respectively. Click here for file

Additional file 4 **Network 2 from gene cluster 3** The top functions of this network are Infection mechanism, gene expression, and cell death. This network is focused on NF-κB molecule (purple circle). Canonical pathways and functions are overlaid with red and yellow edges, respectively. Click here for file
