# Supplementary material for: Gene expression pattern in swine neutrophils after lipopolysaccharide exposure: a time course comparison
Source: BMC Proc. 2011 Jun 3;5(Suppl 4):S11. doi: 10.1186/1753-6561-5-S4-S11 (PMC3108205; doi:10.1186/1753-6561-5-S4-S11)
Supplement: Additional file 1 — IPA Networks Networks are selected if their scores are higher than 25 for each cluster (except for cluster 1, because the number of genes is too small to analyze with IPA). The table contains columns with the network number, the name of the comparison list, names of the genes involved in the network, the score value and the top functions. Focus genes are shown in bold. [file 1753-6561-5-S4-S11-S1.pdf]

Supplemental Data 1: IPA networks

Blue – Cluster 2
Yellow – Cluster 3

ID	Analysis	Molecules in Network	Score	Focus Molecules	Top Functions
1	Cluster 2	**ACE2,** Alp, **ARFGAP3, ATF2, BAZ1B, BAZ2A, BCS1L, C5ORF34, COL24A1, COPS6, CSF1, DBF4, DDX24, ECH1, ELP2, ELP3 (includes EG:55140), FANCC, FANCM, GLS,** HISTONE, Histone h3**,** Histone h4, Ifn, IFN alpha/beta, IFN Beta, IFN TYPE 1, IgG, IL1, IL12 (complex), IL12 (family), Il12 receptor, Immunoglobulin, Interferon alpha, **INTS12,** JAK, **MAP2K6, MBOAT2,** MHC Class II (complex), **MICAL2, NCOA1, NMT1, NPM1 (includes EG:4869), OLFML3, ORC2L, RAP1GDS1, RBBP7, RNF10, RNF138, Rnr, RPA1, SEC31A, SERPINB9, SET, SETDB1, SIN3A, SLC39A6, SMARCA1, STAM2, STAT4,** STAT5a/b, **STK40, SYPL1, TAF2, TAF5, TAOK1, TAX1BP1, TMEM167B, TRDMT1, TYK2, UBE2K**	59	51	Cell Cycle, Cellular Assembly and Organization, DNA Replication, Recombination, and Repair
2	Cluster 2	**ACLY,** Actin, **AGK, AHCTF1, C19ORF50, CACNA1C, Caspase, CDC25C,** Cofilin, Collagen type I, **CSE1L,** Cyclin A, Cyclin E, **DDX23, EED, EFTUD2, EIF2A, ELAVL1,** ERK1/2, F Actin, **FAF2,** Histone H1, **HNRNPC, ILF2 (includes EG:3608),** Importin beta, Insulin, **IPO5, IPO7, KLF13, KPNA3, KRAS, LMO3, LRPPRC, NEK1, NUP93, NUP133, NUP214,** PDGF BB, **PFKFB2, PFKFB3,** PP1 protein complex group, PP1-C, PP2A, Pp2c, **PPP1CA,** Ppp2c, **PPP2R1B, PPP2R3A, PPP2R5C, PPP2R5E,** Profilin, **PTBP1,** Rb, **RDX,** Rock, **ROCK1, SEH1L, SFRS7, SFRS12, SH3GLB2 (includes EG:56904), SNRNP40, SOCS6, SRPK1, TIA1, TIAL1, TOM1L1, UBA5, UBR1, XPO5, ZRANB2**	58	50	RNA Post-Transcriptional Modification, Cellular Assembly and Organization, Cancer
3	Cluster 2	**ABCE1, ACSL3,** Adaptor protein 2, **ATF7IP,** ATPase, **BAMBI, CAPRIN1,** Cbp/p300, **CELF2, COL4A3,** Collagen Alpha1, Collagen type III, Collagen type IV, **CREG1,** Ctbp, **CTSK, DCLRE1A,** Dynamin, **E2f, EEA1, EGLN1, ELMO1,** Eotaxin, **ERBB2IP (includes EG:55914), GCC2, GGPS1, GLCE, GOSR1, GRIN2B, IDE,** Integrin alpha V beta 3, **ITGAV, KDELR2, KIF1B, KIF20B, LAMA2, LCOR, MARCH7, MYO9B, MYOF, NOC3L, NSF, PGRMC1,** Pias, **PIAS1, PIAS2, PRPS1, PSMC2, PSME4, RAB6A, RBL1, RBMS1, RYR2, SAR1A, SCFD1, SEC24B,** Smad, **SMAD4,** Smad2/3, Smad2/3-Smad4, Snare, **SPAST, SPTBN1, SUSD1,** Tgf beta, **TGFB1, TTC3, UBE4B, VTI1A, ZNHIT6**	58	53	Molecular Transport, Protein Trafficking, Cellular Development
4	Cluster 2	**ADAM10,AKT3,ALAS1,**alcohol group acceptor phosphotransferase,AMPK,**ARL6IP5,** ATPase, **BAZ1B,C5ORF34,**Calpain,**CDC27,CROT,**Cytochrome c,**EED,EIF2A,**Estrogen Receptor,**FNDC3A,**FSH,**GOPC,**Gsk3,hCG,HISTONE,Histone H1,Histone h3,**IDE,IGF1R,**Insulin,**IPO7,KIF1B,KIF20B,**Lh,**MYO9B,**NADPH oxidase,**NCOA1,NCOA4,NEK2,NEO1,NMT1,NPM1 (includes EG:4869),**peptidase,**PFKFB3,**Pka,Pkc(s),**POLDIP3,PP1-C,PPP1CA,PPP2R1B,PPP2R3A,PPP2R5C,PPP2R5E,PRKAA1,PRKAB2,PTBP1,RAB4A,RANBP3,**Rb,**RCC1 (includes EG:1104),RPL35A,**Rxr,**SEC31A,SET,SMARCA1,SPAST,ST6GAL1,T3-TR-RXR,TRIM24,TRIM33,**Trypsin,Vegf,**XK**	55	46	Cellular Assembly and Organization, Cellular Function and Maintenance, Drug Metabolism
5	Cluster 2	**ADAM10,** Akt, **AKT3,** AMPK, Ap1, **AP1S2,** Ap2 alpha, **AP3B1, AP3S2, BIN1,** Calcineurin A, Calcineurin protein(s), Calmodulin, CaMKII, **CENPC1,** Clathrin, **CLN3,** Creb, Cytochrome c, **DNAJA2, DNAJB12, DNAJC13,** Estrogen Receptor, **FAM48A, FBXO32,** Fibrinogen, Hdac, Hsp27, Hsp70, Hsp90, Hsp22/Hsp40/Hsp90, Jnk, LDL, **LRRK2, MAP4K3, MAPK9, MAPK14, MMP2, MYD88,** N-cor, NGF, **NUMB, OPA1,** P38 MAPK, Pi3-kinase, **PICALM, PPM1D, PPP3CA, PPP3CB, PRKAA1, PRKAB2, PRKCB, PTRF, RAC2,** Rar, Rxr, Sapk, **SYN2, SYNJ1, TACC1,** Tnf, Tnf receptor, **TRAF5, TRIM24, TRIM33,** Tubulin, Vegf, **VEGFA, XPOT, ZNF148**	38	39	Cellular Assembly and Organization, Cellular Function and Maintenance, Cellular Movement
6	Cluster 2	**ACLY, ARMC1,** C14ORF1, **C19ORF42, C2ORF47,** CBX5, **CCDC41, CLYBL, DDX10,** DSN1, F9, **GGCX, GMDS, GPC6,** HNF1A, HNF4A, **KBTBD4, KIAA0196, KIAA1704, KLHL20,** KNG1 (includes EG:3827), KPNB1, LUC7L2, MIR183 (includes EG:406959), MIR194-1 (includes EG:406969), MIR194-2 (includes EG:406970), MIS12, **MLF1IP, MRP63, MRPL33,** NAA10, **NAA50, NRD1, NSL1, NUP54,** NUP62, NUTF2, **ORMDL1, ORMDL2, PCNP, PEF1, PLEKHA8,** PNKP, **RBKS,** retinoic acid, SEC23A, **SEC23IP,** SFRS1, SFRS2, **SFRS11, SLC25A40, SLC33A1, SLC39A9, SLC7A6OS,** spermine, **TMEM30A,** TRAF6, **TXNL4B,** XPO1	35	35	Cell Morphology, Cellular Assembly and Organization, Gene Expression
7	Cluster 2	APC-FZR1, **ARID1A, ASCC1, ASCC3,** B3GAT1, **B3GAT2,** BCL6, **BMS1, C16ORF5, C19ORF63,** C1ORF9, **C1ORF112,** C20ORF111, **C5ORF15,** CCNB2, CCNB3, CCNB1IP1, Cdc2, Cdc2-CyclinB, Cdc2-CyclinB-Sfn, CDK1-Cyclin B, CHST10, **CNOT1, CNOT2,** Cyclin B, **DCTN6,** EPO, FAM49B, FGF12, galactosylgalactosylxylosylprotein 3-beta-glucuronosyltransferase, GSPT1, HCCS, HCN1, IFT80, IMPDH1, **INTS8, JAZF1**, KCMF1, **LPHN3, LRRC57**, MGAT4A, **MGAT4B,** MIR201 (includes EG:387197), MIR202 (includes EG:387198), MIR298 (includes EG:723832), MIRN330, MYEF2, **MYST4,** NAV1, **NEO1, NKAIN2, NTRK3**, OSBPL1A, **PCDH15, PDS5A, PDS5B, PHF6**, PLXNA2, **PPIP5K2**, REST, SLC10A3, SLITRK2, SRF, STAG1, SYTL3, **TOMM70A,** UBE2Z, **WAPAL, WDR35 (includes EG:57539),** XIAP	30	33	Genetic Disorder, Neurological Disease, Carbohydrate Metabolism
8	Cluster 2	**ABLIM1, ALG2, ARL5A, DDX60, DERL1**, DLG4, DUB, ESR1, **GLE1, GOLPH3L**, HLA-A, IFNA2, **IMPA1,** IRF3, **KLHL9**, KRT10, **LRRC7**, MIRN324, NCK2, **NFX1, REEP5, SEPT11**, SMAD3, SMN1, **STAU2**, TERT, **TOR1A, TOX2, TRAF3, TRIM37, TXNDC11, UBASH3B, UGGT2, USP1**, USP16, USP17, USP19, USP20, USP24, USP26, USP28, USP29, USP30, USP32, **USP33**, USP35, USP36, USP38, **USP40**, USP41, USP42, USP43, USP44, **USP45**, USP46, **USP47**, USP49, USP50, USP53, USP54, **USP21 (includes EG:27005),** USP27X, USP37 (includes EG:57695), USP51 (includes EG:158880), USP9Y, **WBP11**, XBP1, **ZC3H4, ZNF143, ZNF329**	26	31	Post-Translational Modification, Cell Cycle, Cancer

9	Cluster 2	ACAA2, ANTXR1, APH1A (includes EG:51107), APH1B, ART1, ATL3, BMP6, **BTRC, CAND1**, CENPB, CHSY1, Ck2, CLSPN, **CPNE3, CTCF**, CUL3, **CYP1B1**, DTNB, **ELOVL5, FAF1**, FAM57A, **FBXW11, GNPDA2, GTF3C2, HADHB, HMG20A**, HSH2D (includes EG:84941), KATNA1, KIF2A, KLHDC5, **KLHL10, LASS2, MIB1**, MIR1, MIR124, MIR155 (includes EG:406947), **NOLC1**, PARP, **PARP1, PARP2**, PARP3, PARP4, PARP6, PARP8, PARP9, PARP10, PARP11, PARP12, PARP14, PARP15, PARP16, PER3, PSENEN, **QSER1, RABGAP1L**, RBM47, **RCBTB1**, RIPK4, **SLC18A2, SLC25A30, SLC39A10**, SNCAIP, **SSB, TIPARP, TM6SF1**, TRAF1, TRIB2, **TWF1, XK, XPNPEP3**	26	30	Amino Acid Metabolism, Post-Translational Modification, Small Molecule Biochemistry
10	Cluster 2	14-3-3, **ARHGAP1, ASAP1, CAMK4, CBLB, CD2**, CD3, CD8, **CD3D, CD3E, CD3G, CFLAR**, ERK, Fcer1, Focal adhesion kinase, **FUCA1**, Gap, **GCH1, HAVCR1**, Ige, IKK (complex), Importin alpha, Integrin, MAP2K1/2, MAP3K, **MAP3K4**, Mapk, Mek, MKK3/6, **MSN, MTSS1, NCF2**, NCK, NFAT (complex), Nfat (family), NFkB (complex), p85 (pik3r), Pak, Pdgf, Pdgfr, PI3K, **PIK3R1**, Pkc(s), PLC gamma, PP1/PP2A, Ptk, **PTPN22**, Rac, **RAF1**, Rap1, **RAP1A**, Ras, Ras homolog, **RASA1, RASA2**, Rho gdi, **RIOK3, RSU1, SCP2, SOCS4**, Sos, **SOS1**, SYK/ZAP, TCR, **TEC, TFG, TRGV9**, VAV, **VPS36, WIPF1**	25	32	Cellular Function and Maintenance, Hematological System Development and Function, Immunological Disease
1	Cluster 3	14-3-3, Actin, **AKAP7, AKAP13**, Alpha catenin, Alpha tubulin, **BRD7, C7ORF42, CAND1, CD164, CDH11, CENPJ, CREBZF, CSDE1, CYFIP2 (includes EG:26999), EPB41, ERG**, F Actin, **FBXW7**, FSH, **GIPC2**, Importin beta, **IPO5, KCNQ1**, Lh, **MDFIC**, MIR1, MIR124, **MYH9, NPM1 (includes EG:4869), NUP214, OLFML3, PIK3R1**, Pka, Pka catalytic subunit, **PLS1, PRKAR2B**, Profilin, **PTBP1**, Ptk, **QSER1, RAB4A, RAB8A, RABGAP1L, RAC2**, Ras homolog, **RDX, RHOJ, RIMS2**, RNA polymerase II, **RPL8, RPLP1, SETDB1, SLC25A30, SMAD4, SNX3, SPTBN1, SRP19, SUV39H2, TCEA1, TCERG1, TEC, TLK1, TRIM24, TRIM33, TWF1, UTRN, WIPF1, XPNPEP3, XPO5**	67	54	Gene Expression, Molecular Transport, Protein Synthesis
2	Cluster 3	19S proteasome, 20s proteasome, 26s Proteasome, **BCAR3**, BCR, **CCDC90B**, Ctbp, **DAB1, DTX3L, EIF2AK2, HIPK2**, Hsp90, Ifn, IFN TYPE 1, Ikb, IKK (complex), Ikk (family), Il12 receptor, JAK, **JAK1, LCOR, MAP3K7**, NFkB (complex), Pias, **PIAS1, PIAS2, POLK, PRDX5, PSMA7, PSMC2**, PSMD, **PSMD1, PSMD2, PSMD10, PSMD12, PTPLAD1, RAD18, REV1, RFWD3, RIOK3, RNF10, RNF14, RNF31, RNF138, ROCK1, RSAD2, SCP2**, SOCS, **STAM2, TAC3, TAOK1, TFG**, Tnf receptor, **TRAF3, TRAF5, TRIM26, TRIM27, TRIM37, TYK2, UBA6, UBAP2L**, UBE2, **UBE2D2, UBE2E3, UBE2J1, UBE2K, UBE2V1, UBE3A**, Ubiquitin, **ZBP1**	59	50	Infection Mechanism, Gene Expression, Cell Death
3	Cluster 3	AChR, **ALG5, ARID4B**, Caveolin, Cbp/p300, **CCDC76, CDHR1**, Collagen Alpha1, Cyclin A, Cyclin B, Cyclin E, E2f, **EP400, EXT1, FSCN1**, Hdac, **HDAC7**, HISTONE, Histone h4, **KIF20A, LTBP1, MXD4**, N-cor, **NCOR1, NR2C2, NSF, NTHL1, NUCB2, OSBPL3, PAX2, PGRMC1, PRPS1, PSME4, RAB6A, Rar, Rb, RBBP7, RBL1, RSU1, RYR2, SAR1A, SEC24B, SEC61B, SEC61G, SERTAD1, SFXN1, Sin3A, SIN3A**, Smad, Smad2/3, Smad2/3-Smad4, **SMC3**, Snare, **SPEN (includes EG:23013), STAG1, STX6, STXBP1, STXBP5**, Syntaxin, Tgf beta, **TGFB1**, TIP60, **TRDN, TRIM9, UBE4B, VAPB, VTI1A, WDR76, YEATS4, ZNHIT6**	48	48	Cellular Assembly and Organization, Tissue Morphology, Cell Cycle
4	Cluster 3	Alp, **CD163, CXORF15, FDX1**, Histone h3, **HMGB1 (includes EG:3146)**, Hsp27, Hsp70, **HSPA6, IFI44L**, IFN alpha/beta, IFN Beta, Ifn gamma, Ige, IgG, IL1, IL12 (complex), IL12 (family), Il18r, **IL18R1**, Immunoglobulin, Interferon alpha, **INTS12**, IRG, **ISG15**, Jnk, LDL, **LTB, MBOAT2**, MHC Class II (complex), MKK3/6, Mre11, **MX1, NEIL1, NKTR, OAS1, ORC2L**, P38 MAPK, **PARP, PARP1, PARP2, POLB, POT1, PPM1D, PTRF, RAD50, RAP1GDS1, RPA1, SELENBP1, SERPINB9, SET, SLC39A6, SP100, STAT4, SYPL1**, Taf, **TAF2, TAF5, TAF15, TCP1, TERF2**, TFIIA, **TIPARP, TMEM167B**, Tnf, **TNKS, TOP3A, TRIM21, ZC3HAV1, ZNF148**	47	44	Cellular Assembly and Organization, Cellular Function and Maintenance, DNA Replication, Recombination, and Repair
5	Cluster 3	AFG3L1, ALB, ATF7IP, ATP11B, ATP13A2, ATP13A5, ATP5S, ATPase, BMYC, **C12ORF35, C14ORF102, CCDC14, CCDC56, CHMP4A, CNOT1**, DAPK1, DDX3Y (includes EG:26900), DQX1, **ENOX2, EXPH5, FAM113B, FOXN4, GLE1, HINT2**, HSPA5, ITFG1, KATNA1, **KATNB1, KIAA1486, KRIT1**, KRT10, MDN1 (includes EG:362498), MIR34A (includes EG:407040), MIRLET7F2, MMP14, **NOTCH2NL**, NR4A2, NTP, **NXT2, PANK2, PHF6, RALGPS2, RANBP3**, RET, RNPS1, **RSRC2**, RYR1 (includes EG:6261), **SCARA3, SECISBP2, SELT, SFRS16, SIL1, SKIV2L**, SMAD3, SMAD9, **SPAST, SSB, SUSD1, TLN2, TOMM7, TOMM70A, TTC3**, UBQLN4, UCHL5, VPS4B, **YIPF6**, YWHAZ, **ZC3H13, ZFP91, ZNF329**	41	40	Nervous System Development and Function, Tissue Development, Tissue Morphology
6	Cluster 3	Adaptor protein 2, **AGK**, Ap2 alpha, **BCL2**, Calcineurin A, Calcineurin protein(s), Calmodulin, Caspase, CD3, Ck2, Clathrin, **CLTC, CRK**, Cytochrome c, **DAB2, EFTUD2, EPHB2**, ERK1/2, Fcer1, FCGR1A/2A/3A, Focal adhesion kinase, Gap, **GATA2, GLRA2**, GNRH, Mek, **MGMT**, Mlc, NCK, NFAT (complex), Nfat (family), **NUCKS1, NUMB, OPA1**, Pdgfr, **PHF21A, PICALM**, Pkc(s), Pkg, Pp2b, **PPP3CA, PPP3CB, PRKCB, PTPN22, RAF1, RAP1A, RASA1, RASA2, RGS3, SCG2, SCYL2, SFRS7, SFRS12, SGPL1, SH3GLB2 (includes EG:56904), SOCS6**, Sos, **SOS1, SRPK1, SUB1**, SYK/ZAP, **SYN2, SYNJ1**, TCR, **TOM1L1, TRA2A, TRGV9**, Tubulin, **UBA5, ZRANB2**	35	41	RNA Post-Transcriptional Modification, Cancer, Cell Death
7	Cluster 3	**ARMC1**, ATF7, BCL6, BCL2L1, **C19ORF42, C19ORF61, C2ORF47, CCDC25**, CCND1, CD80, CDKN1B, **CWC25, DACH2**, DUSP6, EHMT1, **EIF1AD**, EIF4ENIF1, EP300, EPO, ERCC5, **FBXO31 (includes EG:79791), GPKOW**, GSTK1, HDAC4, HNF1A, HNF4A, JUNB, **KIAA0196**, MED23, MIRN323, **NBAS, NCOR1**, NCOR2, **OGFR**, ONECUT1, **ORMDL1, ORMDL2, PCNP, PLEKHA8**, POLRMT, PPARD, PPARGC1A, **PPIP5K2, RBKS, RNF113A**, SDC1, SEC23A, **SEC23IP**, SERPINE1, **SLC25A40, SLC33A1, SLC7A6OS**, SMAD3, **SMAD4, SORCS3**, SP1, STAT1, **STAU2, TFB1M, TMEM30A**, TNFRSF14, TNFSF13, **TRIM4**, TSN, **TTC37, TXNL4B, UTP23, WDR35 (includes EG:57539)**, ZNF443	34	35	Gene Expression, Cell Cycle, Digestive System Development and Function

8	Cluster 3	Akt, AMPK, Ap1, **CNTNAP2, COL4A1,** Collagen type I, Collagen type IV, Creb, **CYTH1, CYTH3, DBI, EIF2A, EPAS1,** ERK, Estrogen Receptor, **FOSB, FOXO1, FURIN,** G protein alphai, Gm-csf, **GSS,** hCG, Histone H1, **IGF2,** Insulin, Integrin, **LSM5,** MAP2K1/2, Mapk, **MPDZ, NEK1,** Neurotrophin, **NRG1,** p70 S6k, p85 (pik3r), Pdgf, PDGF BB, **PFKFB2, PFKFB3, Pi3-kinase, PI3K, PLAUR, PLC gamma, POLDIP3,** PP1 protein complex group, PP1-C, PP1/PP2A, PP2A, **PPP1CA,** Ppp2c, **PPP2R1B, PPP2R3A, PPP2R5C, PPP2R5E,** PRKAA, **PRKAA1, PRKAB2, PRRX1,** Rac, Rap1, Ras, Shc, **SMG5, SNRPE, SNRPG, SOCS4,** STAT5a/b, **TACC1,** Vegf, **VEGFA**	29	34	Cell-To-Cell Signaling and Interaction, Respiratory Disease, Cardiovascular System Development and Function
9	Cluster 3	4933405O20RIK, 9130409I23RIK, **ATAD2,** BAX, BMP6, **CCDC11,** CCDC85B, CDKN1A, **COX1, COX6B1,** COX6B2, COX7B2 (includes EG:170712), **COX7C (includes EG:1350),** COX8C, CXORF21, CYB5RL, CYP4EA, Cytochrome c oxidase, **DDX60, DHRS13,** DHRSX, DUB, ERRFI1, ESR1, **FBRSL1,** GFOD2, GYKL1, HLA-A, IFNA2, **LONP1,** MIR26A1, **NT5E, NUP54, OSBPL9,** oxidoreductase, OXNAD1, **PDXDC1,** progesterone, **RDH13, RNF213, SLC18A2, SLC39A10, SOSTDC1,** SVS1, **UBA3, UGGT2, USP1, USP16,** USP17, USP26, USP29, USP30, USP32, **USP33,** USP35, USP36, USP38, **USP40,** USP41, USP42, USP43, USP44, **USP45**, USP47, USP50, **USP21 (includes EG:27005),** USP27X, **USP37 (includes EG:57695),** USP51 (includes EG:158880), **VGLL4**	25	29	Behavior, Reproductive System Development and Function, Reproductive System Disease
10	Cluster 3	3-hydroxybutyric acid, 9330129D05RIK, ACAD8, ACAD9, ACAD10, ACAD11, ACADS, ACADSB, acyl-CoA dehydrogenase, ANO4, ARFGAP3, ATP2C1, **AZGP1,** C9ORF125, Ck2, **DPM1, FAM104A,** FAM38B, GART, **GCDH, GOLPH3L,** IFNG, INSRR, IVD, **IWS1,** LINGO2, LMAN2, MIR210 (includes EG:406992), MIRLET7A1, MIRLET7B (includes EG:406884), MOGS, MYC, NSUN3, **NUP93, NUP133,** NUP205, **PCM1, PCNT, PEX2,** PEX10, **PEX19, PHF8,** PNN, PTGES2, RT1-B, **SEC11C, SEC16B, SEH1L, SFRS18,** SLC11A1, **SLC38A9, SLC39A9,** SRP54, **SUGT1, TIA1, TIAL1,** TMEM178, **TUBGCP2,** TUBGCP3, **TXNDC11, ULK3,** XBP1, XIST, **XK, XPOT,** ZCCHC3, ZCCHC14**, ZNF143, ZNF281,** ZNF462	25	29	Cellular Assembly and Organization, Cellular Development, Hematological System Development and Function
