# Supplementary material for: Gene expression pattern in swine neutrophils after lipopolysaccharide exposure: a time course comparison
Source: BMC Proc. 2011 Jun 3;5(Suppl 4):S11. doi: 10.1186/1753-6561-5-S4-S11 (PMC3108205; doi:10.1186/1753-6561-5-S4-S11)
Supplement: Additional file 2 — IPA Biofunctions and Canonical Pathways Top 10 IPA biofunctions and canonical pathways. a) Biofunctions: Tested by the Fisher Exact test p-value. b) Canonical Pathway. For each list, the pathways are ranked by the score [score = -log(p-value)] using the same criterions than biofunctions. The table includes the ratio (number of focus molecules in a given pathway divided by the total number of the molecules that conform that pathway) [file 1753-6561-5-S4-S11-S2.pdf]

Supplemental Data 2:

a) IPA Biofunctions

Cluster 2	P-value
Molecular Transport	1,16E-06-3,22E-02
Protein Trafficking	1,16E-06-1,24E-02
Post-Translational Modification	3,66E-06-2,15E-02
Protein Degradation	6,34E-06-2,09E-03
Protein Synthesis	6,34E-06-2,59E-02
Cell Cycle	7,33E-06-3,65E-02
RNA Post-Transcriptional Modification	8,74E-06-7,68E-03
Cancer	4,85E-05-3,42E-02
Cellular Assembly and Organization	5,08E-05-3,22E-02
Cellular Function and Maintenance	6,49E-05-3,48E-02
Cluster 3	P-value
Cell Morphology	4,73E-06-3,5E-02
Dermatological Diseases and Conditions	1,14E-05-3,5E-02
RNA Post-Transcriptional Modification	1,22E-05-3,5E-02
Gene Expression	1,63E-05-3,5E-02
Infectious Disease	2,53E-05-2,29E-02
Developmental Disorder	2,68E-05-3,5E-02
Renal and Urological Disease	3,15E-05-4,65E-05
Cell Death	3,77E-05-3,5E-02
Infection Mechanism	3,84E-05-3,5E-02
Cellular Assembly and Organization	4,98E-05-3,5E-02

b) IPA Canonical Pathways

Cluster 2	-log(p-value)	Ratio
JAK/Stat Signaling	5,77E00	1,88E-01
Cdc42 Signaling	5,62E00	1,27E-01
Protein Ubiquitination Pathway	5,12E00	1,04E-01
VEGF Signaling	4,6E00	1,34E-01
CTLA4 Signaling in Cytotoxic T Lymphocytes	4,54E00	1,33E-01
Regulation of IL-2 Expression in Activated and Anergic T Lymphocytes	4,39E00	1,33E-01
Regulation of eIF4 and p70S6K Signaling	4,37E00	1,06E-01
RANK Signaling in Osteoclasts	4,04E00	1,25E-01
Production of Nitric Oxide and Reactive Oxygen Species in Macrophages	3,59E00	8,65E-02
T Cell Receptor Signaling	3,55E00	1,12E-01
Cluster 3	-log(p-value)	Ratio
Protein Ubiquitination Pathway	7,17E00	1,19E-01
JAK/Stat Signaling	5,12E00	1,72E-01
IL-3 Signaling	3,19E00	1,25E-01
PDGF Signaling	2,96E00	1,18E-01
Pancreatic Adenocarcinoma Signaling	2,95E00	9,48E-02
PI3K/AKT Signaling	2,86E00	8,63E-02
Chronic Myeloid Leukemia Signaling	2,78E00	9,52E-02
AMPK Signaling	2,72E00	7,88E-02
Molecular Mechanisms of Cancer	2,67E00	6,18E-02
Ceramide Signaling	2,65E00	1,03E-01
