# Supplementary figures and images for: Gene expression pattern in swine neutrophils after lipopolysaccharide exposure: a time course comparison
Source: BMC Proc. 2011 Jun 3;5(Suppl 4):S11. doi: 10.1186/1753-6561-5-S4-S11 (PMC3108205; doi:10.1186/1753-6561-5-S4-S11)

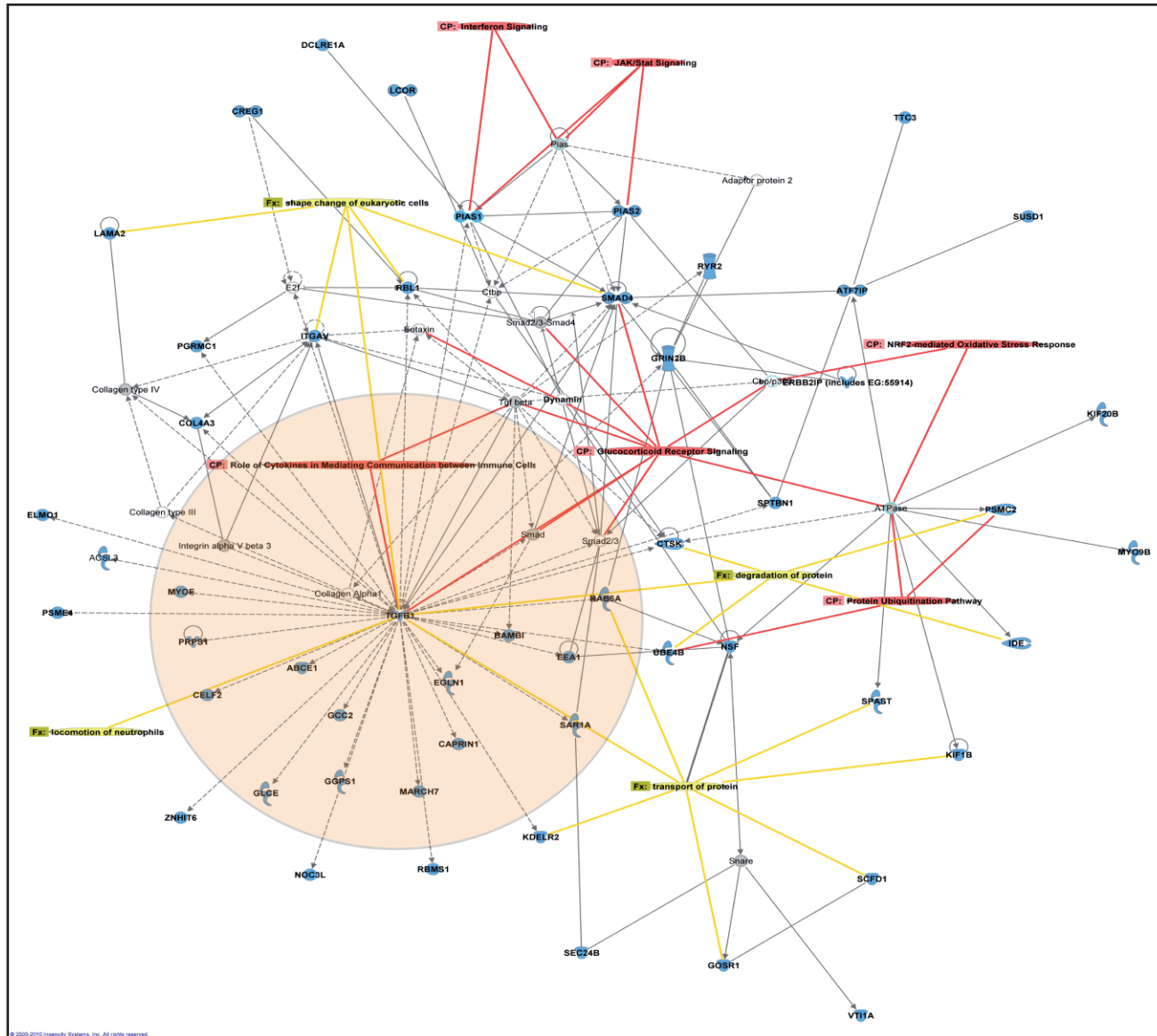

Supplement: Additional file 3 — Network 3 from gene cluster 2 The top functions of this network are Molecular transport, protein trafficking and cellular development. This network is focused on TGFB1 molecule (orange circle). Canonical pathways and functions are overlaid with red and yellow edges, respectively. [file 1753-6561-5-S4-S11-S3.pdf]

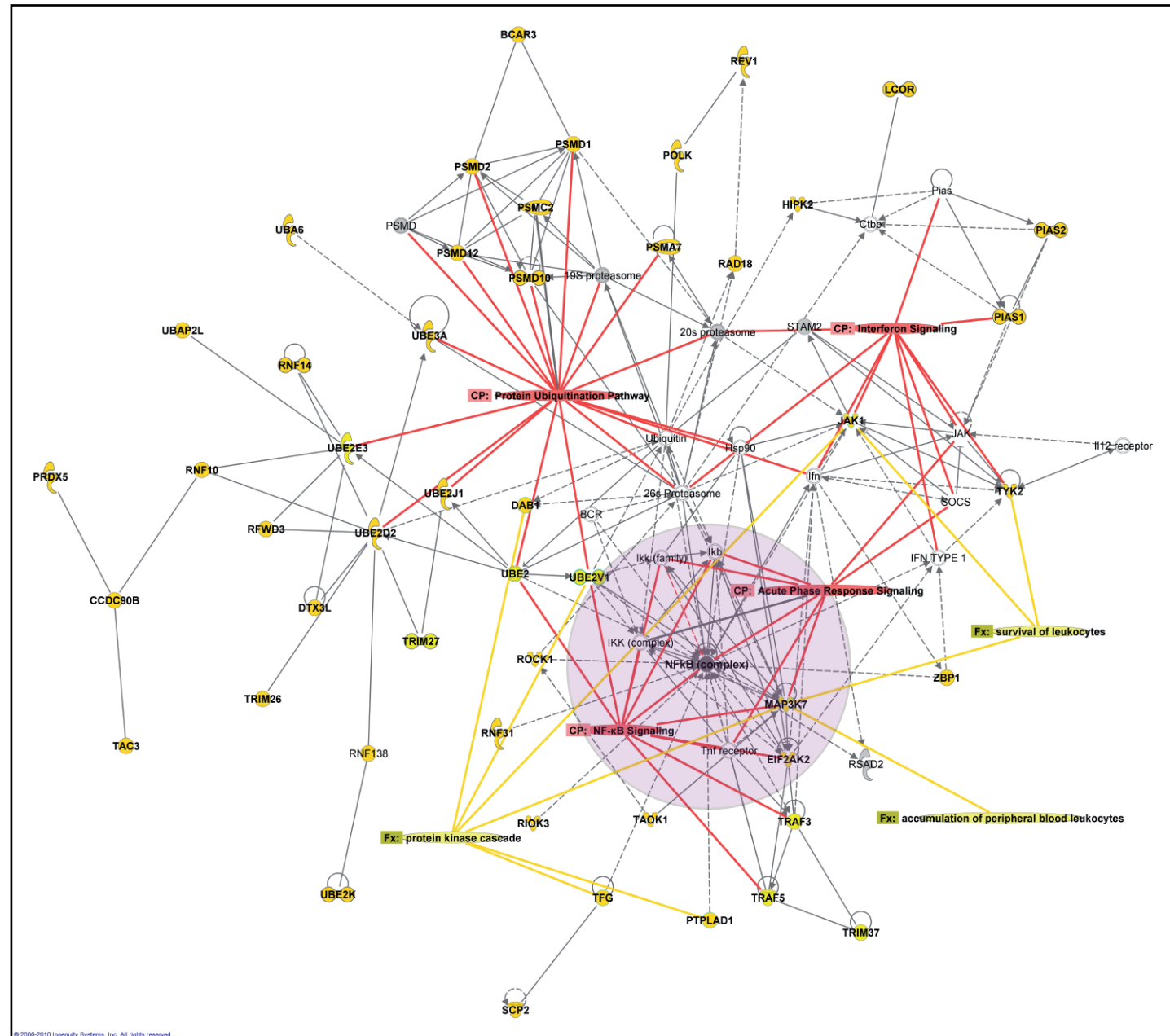

Supplement: Additional file 4 — Network 2 from gene cluster 3 The top functions of this network are Infection mechanism, gene expression, and cell death. This network is focused on NF-κB molecule (purple circle). Canonical pathways and functions are overlaid with red and yellow edges, respectively. [file 1753-6561-5-S4-S11-S4.pdf]
